# Machine Learning in the Classification of Pediatric Posterior Fossa Tumors: A Systematic Review

**DOI:** 10.3390/cancers14225608

**Published:** 2022-11-15

**Authors:** Alexander G. Yearley, Sarah E. Blitz, Ruchit V. Patel, Alvin Chan, Lissa C. Baird, Gregory K. Friedman, Omar Arnaout, Timothy R. Smith, Joshua D. Bernstock

**Affiliations:** 1 Harvard Medical School, Boston, MA 02115, USA; 2 Department of Neurosurgery, Brigham and Women’s Hospital, Harvard Medical School, Boston, MA 02115, USA; 3 Department of Mechanical Engineering, Massachusetts Institute of Technology, Cambridge, MA 02139, USA; 4 Division of Gastroenterology, Hepatology and Endoscopy, Brigham and Women’s Hospital, Harvard Medical School, Boston, MA 02115, USA; 5 Department of Neurosurgery, Boston Children’s Hospital, Harvard Medical School, Boston, MA 02115, USA; 6 Division of Pediatric Hematology and Oncology, Department of Pediatrics, University of Alabama at Birmingham, Birmingham, AL 35294, USA; 7 Comprehensive Cancer Center, University of Alabama at Birmingham, Birmingham, AL 35294, USA

**Keywords:** posterior fossa tumor(s), neuro-oncology, artificial intelligence (AI), machine learning, neuroradiology

## Abstract

**Simple Summary:**

Diagnosis of posterior fossa tumors is challenging yet proper classification is imperative given that treatment decisions diverge based on tumor type. The aim of this systematic review is to summarize the current state of machine learning methods developed as diagnostic tools for these pediatric brain tumors. We found that, while individual algorithms were quite efficacious, the field is limited by its heterogeneity in methods, outcome reporting, and study populations. We identify common limitations in the study and development of these algorithms and make recommendations as to how they can be overcome. If incorporated into algorithm design, the practical guidelines outlined in this review could help to bridge the gap between theoretical algorithm diagnostic testing and practical clinical application for a wide variety of pathologies.

**Abstract:**

*Background*: Posterior fossa tumors (PFTs) are a morbid group of central nervous system tumors that most often present in childhood. While early diagnosis is critical to drive appropriate treatment, definitive diagnosis is currently only achievable through invasive tissue collection and histopathological analyses. Machine learning has been investigated as an alternative means of diagnosis. In this systematic review and meta-analysis, we evaluated the primary literature to identify all machine learning algorithms developed to classify and diagnose pediatric PFTs using imaging or molecular data. *Methods*: Of the 433 primary papers identified in PubMed, EMBASE, and Web of Science, 25 ultimately met the inclusion criteria. The included papers were extracted for algorithm architecture, study parameters, performance, strengths, and limitations. *Results*: The algorithms exhibited variable performance based on sample size, classifier(s) used, and individual tumor types being investigated. Ependymoma, medulloblastoma, and pilocytic astrocytoma were the most studied tumors with algorithm accuracies ranging from 37.5% to 94.5%. A minority of studies compared the developed algorithm to a trained neuroradiologist, with three imaging-based algorithms yielding superior performance. Common algorithm and study limitations included small sample sizes, uneven representation of individual tumor types, inconsistent performance reporting, and a lack of application in the clinical environment. *Conclusions*: Artificial intelligence has the potential to improve the speed and accuracy of diagnosis in this field if the right algorithm is applied to the right scenario. Work is needed to standardize outcome reporting and facilitate additional trials to allow for clinical uptake.

## 1. Introduction

Brain tumors are the second leading cause of death in children under 15 with an estimated incidence of 2–3.5 per 100,000 [1,2]. Posterior fossa tumors (PFTs) comprise 50–74% of childhood brain tumors, with the majority being juvenile pilocytic astrocytomas, medulloblastomas, ependymomas, and brainstem gliomas [3,4]. Central nervous system tumors in the pediatric population frequently present with nonspecific symptoms, which can lead to delays in diagnosis and treatment. One study found that the average time to diagnosis in a cohort of pediatric brain tumor patients was 7.7 months after symptom onset [5]. Given the rapid progression of some pediatric brain tumors, delays in diagnosis are associated with significant morbidity and mortality. Since treatment varies based on the type and grade of PFT, it is imperative to obtain an early diagnosis in this highly morbid group of malignancies. Histopathological diagnosis remains the standard of care for the diagnosis of PFTs. While accurate, this method is time consuming and requires a tissue specimen as well as access to a trained neuropathologist. While conventional magnetic resonance imaging (MRI) can be used to evaluate tumor location and impact on surrounding structures, it is of limited diagnostic value. Radiological differentiation between different PFTs is difficult and can be further complicated by tumor mimics such as demyelinating disorders and Alexander disease [6].

Some progress has been made to improve the diagnostic accuracy of imaging with the addition of advanced MR sequences such as diffusion-weighted imaging (DWI). Using apparent diffusion coefficient (ADC) ratios, radiologists in one study were able to discriminate pilocytic astrocytomas from ependymomas with a sensitivity of 83% and a specificity of 78% [7]. The discovery that individual radiomic and molecular features correlated to distinct PFTs led to the application of artificial intelligence for the diagnosis and subclassification of these tumors. Prior work has shown that artificial intelligence is becoming an increasingly viable tool with the potential to improve diagnostic speed and accuracy [8,9]. Machine learning has already been heavily implemented in the diagnosis of brain tumors in both children and adults, with previous studies reporting algorithms that can differentiate gliomas, meningiomas, and pituitary tumors based on extracted imaging features with accuracies as high as 99% [10,11,12]. Additional work has shown the possibility of using these methods to not only differentiate between tumor types, but also to subclassify tumors by grade, stage, and even molecular features [12,13,14]. Similar methods are now being explored to diagnose and classify PFTs. In this systematic review and meta-analysis, we aim to identify and critique all the primary literature that applies machine learning to the diagnosis and classification of pediatric PFTs. We analyze the algorithm architecture and efficacy as well as study parameters, strengths, and limitations to assess the clinical readiness of such technology, provide recommendations of best practices, and highlight areas for improvement. This work serves as a case study on how machine learning classification algorithms can be applied to clinical diagnosis with recommendations that can be applied to other pathologies.

## 2. Materials and Methods

This systematic review of the literature was completed according to the Preferred Reporting Items for Systematic Reviews and Meta-Analyses (PRISMA) guidelines [15]. Standardized electronic searches were conducted in PubMed, EMBASE, and Web of Science to identify relevant articles. Searches were conducted using conjugated “AND” and “OR” statements with keywords related to machine learning, artificial intelligence, and pediatric PFTs (Supplementary). Searches included all articles in the English language from database inception to 31 July 2022.

### 2.1. Inclusion and Exclusion Criteria

All observational studies, clinical trials, case reports, and technical papers assessing the use of machine learning to diagnose or classify PFTs based on molecular or radiomic features were included. No limit was placed on sample size or timeframe. Review articles, abstracts, conference abstracts, and primary papers that did not study the application of a machine learning algorithm (MLA) to the diagnosis or classification of a pediatric PFT met the exclusion criteria. Papers that specifically subclassified pediatric PFTs by other criteria, such as prognosis, response to treatment, etc., were also excluded.

Studies identified by the literature search were screened in two rounds, with the evaluation of appropriateness determined by consensus of the authors. Initially, title and abstract screening was conducted. Papers that met the exclusion criteria were excluded, and then a similar process was repeated with a full text review. Authors resolved all disagreements by consensus.

### 2.2. Data Extraction

Two authors independently extracted full texts of included articles into a standardized extraction table. Disagreements were decided by a two-author consensus. Data collected from each study covered study parameters including title and author, population size by tumor type, tumor type(s) being studied, study location(s), study timeframe, and ground truth used; algorithm parameters including type of input data, training set size, validation set size, test set size, method of image segmentation (manual vs. automatic), normalization used, presence/absence of texture analysis, deep learning model architecture, presence/absence of feature selection, and number of features extracted in final algorithm; algorithm performance statistics including sensitivity, specificity, accuracy, area under the curve (AUC), F1-score, Dice coefficient, positive predictive value, and negative predictive value; comparisons and analyses performed including comparison of the algorithm to a neuroradiologist, neuropathologist, or other clinical standard of care as well as the outcome of the comparison; and both algorithm as well as study limitations.

### 2.3. Gold Standard Comparison

For each paper that included a comparison of an MLA to a gold standard, the minimum and maximum AUCs or accuracies were collected for each method. The following calculations were conducted to compare the best- and worst-case efficacy of each diagnostic method: the difference between the maximum accuracy/AUC for the MLA and the minimum accuracy/AUC for the gold standard was computed. The same calculation was repeated with the maximum accuracy/AUC for the gold standard and the minimum accuracy/AUC for the MLA.

## 3. Results

### 3.1. Search Results

**Figure 1:**
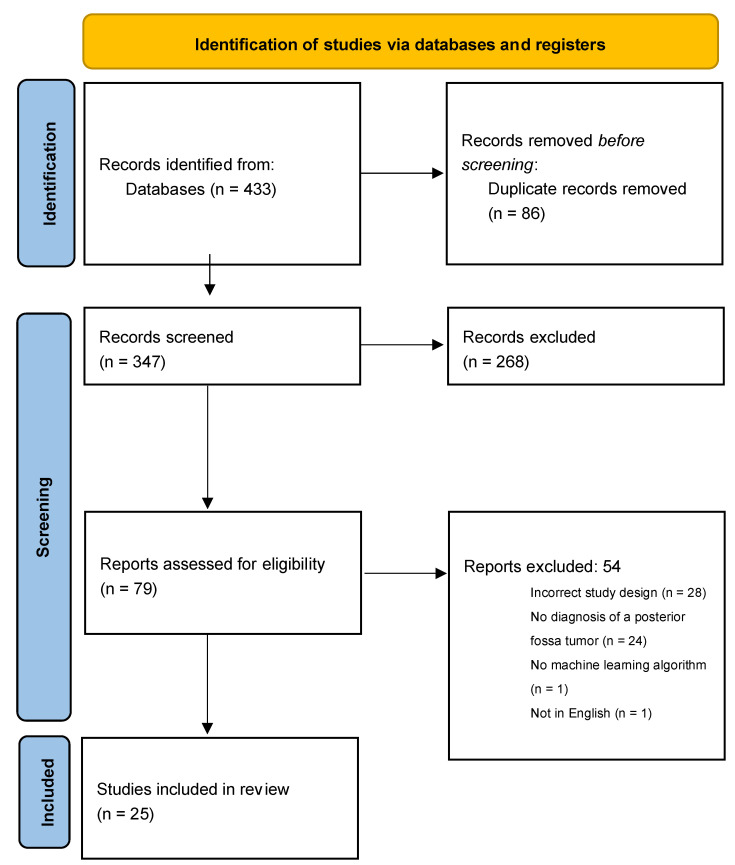
PRISMA flow diagram of study selection [15].

The electronic literature search identified 433 studies, of which 86 were duplicates. Of the 347 records that underwent title/abstract screening, 268 were excluded for irrelevance. The full texts of 79 articles were reviewed, yielding 25 studies that met the inclusion criteria. Of the 54 articles that were excluded, 28 had the incorrect study design, 24 did not diagnose or classify a pediatric PFT, one did not feature an MLA, and one was not in the English language (Figure 1).

### 3.2. Algorithm Study Parameters and Design

Table 1 features study method data from all 25 studies of the MLAs applied to the classification of pediatric PFTs. Twenty-two papers used imaging data to classify PFTs, including both non-contrast and contrast-enhanced T1/T2-weighted MRI, DWI, and MR-spectroscopy [16,17,18,19,20,21,22,23,24,25,26,27,28,29,30,31,32,33,34,35,36,37]. Three papers used molecular methods to classify these tumors based on microscopy slides or methylation array data [38,39,40]. The majority of algorithms were applied to retrospectively created datasets, and histologic diagnoses, as determined by a clinical pathologist, were uniformly used as the ground truth. Most studies were conducted with clinical data from a single site with two studies featuring clinical data from up to seven sites [33,34]. Study populations varied significantly, ranging from cohorts of 23 patients to 617 patients [31,40]. Pilocytic astrocytoma and medulloblastoma were the most well-represented PFTs across all reports with inclusion in 19 and 22 studies, respectively [17,18,19,20,21,22,23,24,25,26,27,28,29,30,31,32,34,35,36,37,38,39,40]. Ependymomas, while included in most studies, had a small individual sample size per study, with many analyses including fewer than 20 ependymoma patients in training or validation datasets [16,17,18,21,22,23,27,29,30,32,36]. Less common pathologies, such as embryonal tumors, gangliogliomas, atypical teratoid rhabdoid tumors, and others, were heterogeneously studied and only featured in a small minority of reports [26,28,38].

Most algorithms were developed and executed using a common workflow. Imaging data were segmented to identify regions of interest using a combination of manual and semiautomatic methods. Eighteen of the papers then included a normalization step in which imaging data were standardized to minimize noise [17,18,19,20,21,22,23,25,26,27,28,29,31,33,36,37,39,40]. Features were extracted from the relevant imaging modalities with some papers yielding as few as 13 features while others generated over 11,000 [20,39]. Nineteen studies employed various methods of feature selection to decrease feature dimensionality for the final analysis [19,20,21,22,23,24,25,26,27,29,30,32,33,34,35,36,37,38,40]. A machine learning classifier was then applied to discriminate between tumor types based on extracted features. Some studies, such as Quon et al. [31], used a single MLA as a classifier. Other papers, such as Li et al. [25] or Grist et al. [22], employed ensembles to combine predictions from multiple algorithms. The range of classifier algorithms used included (Table 2): k nearest neighbor (kNN), support vector machine (SVM), neural network (NN), classification and regression tree, extreme learning machine (ELM), naïve Bayesian (NB), random forest (RF), partial least square regression (LSR), and linear discriminant analysis (LDA).

Eight papers did not fully define the training or validation set employed [22,23,26,27,32,36,39,40]. Of those studies that did, most had a significantly larger training set than validation set. Bidiwala et al. [17] and Fetit et al. [21] both utilized cross-validation given their small sample sizes.

### 3.3. High-Yield Features

Individual features important for the discrimination of PFTs were dependent on the dataset of origin. For generic T1- and T2-weighted imaging, extracted texture features were highly discriminative [21,24,25,27,33,34]. Most discriminative features from DWI were generated from ADC maps. These included ADC mean, ADC skewness, ADC energy, ADC entropy, ADC low grey level zone emphasis, and others [19,20,22,23,26,28,29]. For MR-spectroscopy, mean spectra and lipid peaks were the main discriminators [18,30]. For methylation array data, individual CpG islands had the highest discriminative value [38]. For classifiers generated from microscopy data, nuclear density, tumor-associated macrophage density, nuclear compactness, and maximum radius were most important for discrimination [39].

### 3.4. Algorithm Performance

Twenty-three studies reported general algorithm performance metrics citing mainly AUCs, accuracies, sensitivities, and specificities (Table 3 and Table 4, Supplementary Table S1) [16,17,19,20,21,22,23,24,25,26,27,29,30,31,32,34,35,36,37,38,39,40]. Algorithms performed well only when differentiating between two tumor types. Ependymoma and medulloblastoma were moderately well differentiated by machine learning with reported accuracies of 68.6% to 87.2% and with a maximal AUC of 0.92 [19,25,35]. Machine learning was also fairly accurate when differentiating ependymoma and pilocytic astrocytoma [24].

As expected, algorithms tasked with the head-to-head classification of more than two tumors had more variable results. Thirteen studies investigated algorithms that could differentiate ependymoma, medulloblastoma, and pilocytic astrocytoma. Of these, the accuracy ranged from 37.5% to 94.5% depending on the algorithm [17,20,21,22,23,26,27,29,30,32,35,36,37]. Of all the MLAs, PNN had the highest average performance when differentiating these three tumor types with an average accuracy of 89.7% [27]. Individual algorithms outperformed the average with Dong et al. [20] achieving an AUC of 0.94 to 0.98 and Zhang et al. [35] reporting consistently high accuracies across multiple trials, ranging from 82.6% to 94.5%. Hollon et al. [39] utilized a machine learning analysis of 10x microscopy slides to discriminate between 11 tumor types with AUCs of 0.96 to 0.97 and accuracies ranging from 89.4% to 100.0% depending on the method used. Danielsson et al. [38] similarly achieved an accuracy of 98.3% applying machine learning methods to differentiate between six tumors based on Illumina 450K methylation array data.

**Figure 2:**
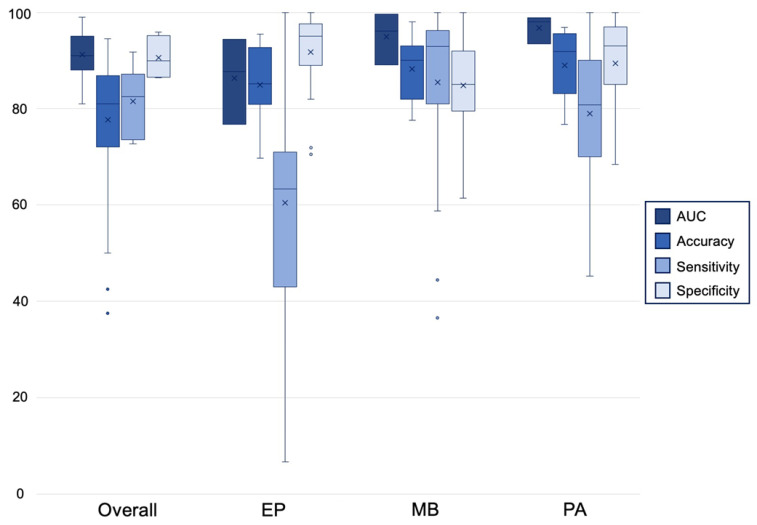
Aggregated algorithmic performance metrics overall and by tumor type. AUCs, accuracies, sensitivities, and specificities are reported for every algorithm developed to discriminate between ependymoma, medulloblastoma, and pilocytic astrocytoma. Parameter means are represented by an “×” and outlier values are illustrated with a “**•**”. If a paper trialed multiple algorithms, each algorithm was individually counted as a separate entry.

The most commonly reported 3-way classifier was between medulloblastomas, pilocytic astrocytomas, and ependymomas (Figure 2). Twelve studies investigated the ability of 50 total algorithms to classify pilocytic astrocytoma [17,20,21,22,23,26,27,28,29,30,35,37]. Diagnostic accuracy ranged from 76.7% to 96.9% [23]. Sensitivities and specificities varied by algorithm, but most algorithms reported both sensitivities and specificities in the 70% to 100% range [17,21,27]. Most of the surveyed MLAs reported similar accuracies in the diagnosis of pilocytic astrocytoma. RF and NB algorithms had the highest mean diagnostic accuracies of 95.8% and 90.7%, respectively, while classification trees had the worst performance with an average diagnostic accuracy of 82.0% [21,26,28].

AUC, area under the curve; EP, ependymoma; MB, medulloblastoma; PA, pilocytic astrocytoma.

Thirteen studies of 64 algorithms quantified their diagnostic performance in the classification of medulloblastoma [17,19,20,21,22,23,26,27,28,29,30,35,37]. MLAs had the best performance in the diagnosis of medulloblastoma with reported accuracies in the 80% to 98% range [21,23]. While some algorithms reported up to 100% sensitivity, others performed poorly with a minimum reported sensitivity of 36.5% [21,27]. Specificities varied by algorithm from 61.4% to 100% [21,27]. While no single MLA definitively outperformed in the diagnosis of medulloblastoma, RF algorithms exhibited the highest mean accuracy of 93.6% [26,28,29].

Fourteen studies of 65 algorithms also quantified the ability of MLAs to correctly diagnose ependymoma [17,19,20,21,22,23,26,27,28,29,30,35,37,40]. Machine learning performed relatively poorly when discriminating ependymomas from other PFTs. While a minority of the developed algorithms achieved accuracies greater than 90%, most reported accuracies around 80% [23,27,35]. Most ependymoma diagnostic algorithms were highly specific but poorly sensitive. One algorithm reported a sensitivity as low as 6.7% with most sensitivities in the 30% to 70% range [21,27,37]. ANNs and classification trees were the most accurate algorithms when diagnosing ependymoma with mean accuracies of 91.5% and 90.0%, respectively [21]. RFs performed inconsistently with an overall accuracy of 81.6% and a standard deviation of 12.0 [19,21,26,28,29].

Leslie et al. [40] reported additional diagnostic accuracies of 85%, 96%, 61%, and 75% for astrocytomas, gliomas, oligodendrogliomas, and gangliogliomas, respectively.

### 3.5. Comparison to Neuroradiologist

**Figure 3:**
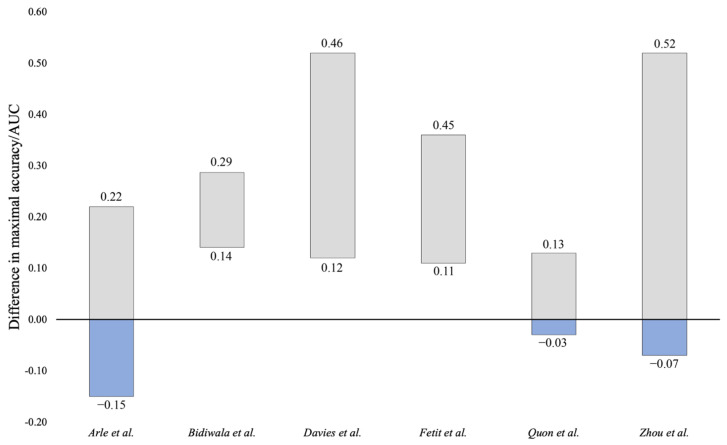
Difference in maximal accuracy or area under the curve (AUC) between machine learning algorithms (MLAs) and neuroradiologists for classification of posterior fossa tumors. The upper value represents the difference between the maximal accuracy/AUC of the MLA and the minimum accuracy/AUC of the radiologist. The lower value represents the difference between the maximal accuracy/AUC of the radiologist and the minimum accuracy/AUC of the MLA. Positive values suggest that the MLA outperformed the radiologist and negative values suggest that the radiologist outperformed the MLA [16,17,18,21,31,37].

The efficacies of the developed MLAs were compared to those of a trained neuroradiologist in seven cases (Figure 3). Algorithms developed by Bidiwala et al. [17], Davies et al. [18], and Fetit et al. [21] all outperformed the neuroradiologist at both best-case and worst-case reported accuracies/AUCs. Of note, Davies et al. [18] was the only study to compare a radiologist to a radiologist augmented by MLA. Results were equivocal for Arle et al. [16], Quon et al. [31], and Zhou et al. [37]. At the maximum reported accuracy/AUC, these algorithms outperformed the standard of care, but at the lower end of reported functioning, these algorithms were inferior to the standard of care at its optimal performance. Payabvash et al. [28] could not be assessed compared to a neuroradiologist because overall accuracy/AUC was not provided for each MLA being evaluated.

### 3.6. Observed Limitations

The limitations of the studied MLAs were divided into methodologic limitations and algorithmic limitations (Table 5). Methodologic limitations relate to study design, the generation of data, and the training of the algorithm. Most major limitations observed were methodologic. Nineteen algorithms (76%) used retrospectively collected data and 18 algorithms (72%) were trained or validated on small samples of fewer than 50 patients, many with incomplete radiographic or molecular datasets [17,19,20,21,23,24,25,26,27,28,29,31,32,33,34,35,37,38,39]. Unequal distribution of tumor types in training sets and the use of the same dataset for both algorithm training and validation were notable limitations. More generally, nine studies provided inadequate descriptions of methods, omitting necessary information such as the training or validation set size, final number of included features, or specific classifier modifications [22,23,26,27,31,32,38,39,40].

Algorithm-specific limitations were less commonly described. Nine algorithms (36%) exhibited significantly worse performance with the classification of a specific tumor histology [17,18,20,21,22,27,31,35,38]. Performance was commonly inferior in the diagnosis of ependymoma due to its limited sample sizes in the training and validation sets [22,27]. Additionally, 24% of studies failed to correlate algorithm output with clinical variables to further improve performance [23,24,25,26,27,31].

## 4. Discussion

In this systematic review, we explored the published literature for MLAs developed to classify pediatric PFTs. Twenty-five studies were identified that applied machine learning approaches to imaging, microscopy slides, and DNA methylation data. In theoretical testing, the MLAs were adept at differentiating PFTs in the pediatric population. Under optimal conditions, individual algorithms achieved AUCs of 0.99 and accuracies up to 100% [21,39]. While medulloblastoma was predicted with the highest accuracy, pilocytic astrocytoma, ependymoma, glioma, ganglioglioma, and oligodendroglioma were also classified with high performance in some cases [17,20,21,22,23,26,27,28,29,30,35,37,40].

Algorithms were heterogeneously crafted and studied. For example, where Arle et al. [16] extracted 20 features to classify 33 tumors using a single NN, Zhang et al. [35] extracted over 1800 features from 527 patients using an ensemble of six different classifiers. Given the vast number of available features to be extracted from multiple data streams, classifier combinations to be applied, and methods of performance analysis to be employed, success in this space depended on the algorithm creators’ ability to select the proper data and methods for the desired goal.

### 4.1. Algorithm Selection

The machine learning approaches employed a variety of classification algorithms to discriminate between PFTs. Surprisingly, while there was some variation, all of the classifiers yielded fairly high accuracies in the individual diagnosis of ependymoma, medulloblastoma, and pilocytic astrocytoma. Instead, there were significant differences observed in the overall accuracy of the MLAs. It is possible that the variation in accuracies reported by studies employing the same MLAs explains some of this discrepancy. Furthermore, algorithm accuracies were only reported on a per-tumor basis in a minority of studies. Studies reporting positive results may be more likely to publish these tumor-specific performance metrics.

LDA, kNN, and RF algorithms had the lowest accuracies with significant variation in the reported results. LDA, while simple to implement, is often critiqued as not being expressive enough to appreciate complex differences between groups [41]. kNN methods, while commonly used, are highly sensitive to dataset size and quality, which may serve to explain the poor performance in the small, unbalanced PFT datasets used in the model training. Additionally, kNN algorithms depend on a knowledgeable operator given the difficulty of choosing a proper k for a given training set [42]. RF models, lauded as a fast ensemble method of classification, are unable to extrapolate datapoints outside the range of the training set and respond poorly to noisy datasets [43]. All three poorly performing algorithms commonly rely upon a broad, high-quality training set, which may have been lacking in these cohorts. While these MLAs have their merits, caution should be employed when applying these methods on small, unbalanced datasets.

The highest-performing 3-way classifiers utilized PNN and NB algorithms. While computationally demanding, PNNs are some of the most effective MLAs in terms of their accuracy and outlier handling [44]. Additionally, PNNs have a history of success in the classification of brain tumors [45]. PNNs are also well-suited to training on a large dataset, compared to other MLAs. NB classifiers are intuitive, scalable, efficient, and robust to outliers. While they assume independence between all features, a higher degree of independence can be insured through the use of feature selection [46]. Both techniques offer the advantages of high accuracy despite the presence of outliers, which may explain their applicability in PFT diagnostics.

With moderate classification accuracies, SVMs were the most frequently employed classifier in this cohort. Given that SVM methods perform well on high dimensionality and unstructured data, such as that derived from imaging, an SVM classifier is a good fit for the PFT classification problem [47]. These benefits come with the associated challenges of long training times and difficulty choosing a proper kernel function [48]. SVM models are additionally known to underperform when trained on datasets that contain significantly more variables than data specimens, which may explain the lackluster results in these cohorts [49].

### 4.2. Objective of Machine Learning Application

Machine learning has generated much excitement as a potential driver of cost reduction and improved diagnostic accuracy in clinical practice. Diagnostic interpretation by a radiologist has previously been shown to be highly operator-dependent, a problem that is further magnified in the diagnosis of PFTs, which have many overlapping radiographic features [50]. Multiple studies have shown that machine learning approaches have improved diagnostic efficacy when compared to their human counterparts [51,52,53]. Imaging-based MLAs applied to glioma diagnosis have shown the potential to improve clinical decision making regarding the diagnosis and management of adult glioma patients [54]. In fact, an artificial intelligence-driven, MRI-based brain tumor diagnostic program has already been integrated into clinical practice with some success [11]. The implementation of a similar platform in the diagnosis of pediatric PFT patients could preclude the need for an invasive biopsy and decrease time to diagnosis. While surgical resection is typically standard of care for these patients, neoadjuvant chemotherapy is sometimes performed [55]. An increased confidence in the diagnosis would allow for the better tailoring of treatment; for example, the importance of obtaining a surgical gross total resection is much greater for improved outcomes in ependymoma compared to medulloblastoma. Finally, the application of MLAs in this space allows for a diagnosis to be obtained in resource-poor settings where a trained neuroradiologist, neurosurgeon, and neuropathologist are not always available.

### 4.3. Translation to Clinical Practice

An algorithm is ready for the clinical environment if it can perform with the equivalent efficacy to the clinical alternative and has demonstrated reliability when applied prospectively in clinic; however, comparison to a clinical standard is often difficult. Given that the standard of care is a pathological diagnosis, little clinical benefit is generated from algorithms that can make a comparable diagnosis based on a tissue sample. Instead, the true clinical value of MLAs is derived from improvements made in the diagnostic accuracy of non-invasive data sources such as imaging. Only seven of the algorithms identified in this review made any performance comparison to a neuroradiologist [16,17,18,21,28,31,37]. Of these, only three definitively outperformed the radiologist. For example, even at the worst-case performance, the algorithm developed by Bidiwala et al. [17] showed a 14% greater accuracy compared to the highest reported accuracy of a neuroradiologist [18,21]. The remaining four studies were more equivocal and developed algorithms that could outperform the radiologist under ideal conditions, but then underperformed in the diagnosis of certain tumor subtypes or when specific classifiers were applied [16,28,31,37]. Given the heterogeneity of developed algorithms in this space, no generalization can be made regarding algorithm performance as compared to a radiologist. However, it seems that under specific conditions, a minority of the posterior fossa classification algorithms can consistently improve the diagnostic accuracy compared to trained neuroradiologists. Unfortunately, no analysis is possible for the molecular diagnostic algorithms given that none of these algorithms were compared to a clinical alternative. In addition, these methods still require a biopsy and no study examined other factors that may justify clinical use, such as improved cost or efficiency compared to diagnosis by a neuropathologist.

Regarding the second standard, a lack of application in the clinical environment is the true barrier to clinical integration of these algorithms. Not a single algorithm from the 25 studies identified in this review was trialed in the clinic. While six algorithms had prospective data collection, they did not apply patient data in real time to yield a diagnosis, as would be expected in a real clinical workflow. Davies et al. [18] took the added step of assessing algorithm performance as an adjunct to a neuroradiologist’s decision making, but this still occurred outside of the clinic. A common critique of MLAs is that the results of theoretical research studies are poorly reproduced when algorithms are used in real time on actual patients [56]. Given the baseline resistance to the clinical uptake of any new technology, such clinical studies are imperative to convince clinicians of the safety and efficacy of these algorithms.

### 4.4. Algorithm Limitations

Limitations in the study and efficacy of these MLAs can be divided into (1) those that are inherent to machine learning methods and (2) those that can be improved with proper study design. Many of the uncontrollable limitations come from the feature extraction stage. Proper feature extraction depends on high signal-to-noise ratios generated from high-resolution imaging. ADC sequences, a common MR-generated sequence used in algorithms classifying PFTs, have inherently lower scan resolution which translates to greater noise, especially when compared to T1- and T2-weighted sequences [31]. Increased noise is difficult to control for and makes the extraction of clinically meaningful imaging characteristics more difficult. Feature extraction from imaging is also limited by the quality of the predominantly manual region of interest delineation and segmentation processes [31]. Seventeen of the included studies featured manual image segmentation with minimal quality control for proper results. Manual segmentation is time consuming and highly operator-dependent, introducing bias into any cohort [57,58]. However, automatic segmentation is not always preferred as it is often ambiguous how the segmentation algorithm defines the region of interest. Finally, the inherent variation between the scans captured by different machines with different calibration methods makes uniform analysis challenging [59]. This limitation is especially relevant to studies that spanned different centers, such as those completed by Zhang et al. [33] or Quon et al. [31], which must contend with sequences captured by different machine makes and models. MLAs rely on minor differences in characteristics between cases to make classification decisions so these minor variations between machines have the potential to alter results.

Many of the methodological limitations commonly observed in machine learning classifiers of PFTs are correctable. The most salient limitation is the small sample sizes used to train and validate these algorithms. Only eight studies reported a training set of over 100 samples and an even fewer three studies reported similarly large validation sets [20,28,31,33,34,35,36,37,38]. This scale (~100 samples) is significantly smaller than that used by the training sets for deep learning models, which typically require at least ~1000 to ~10,000 samples in the supervised setting. Algorithms trained on small sample sizes commonly overfit data, yielding an overestimated accuracy [60]. Furthermore, Bidiwala et al. [17] and Fetit et al. [21] employed a cross-validation method in which one sample was withheld from the training set, the model was trained, and then validation was completed on the single remaining sample. This process was then repeated for all samples and then the results were aggregated. While this is an understandable approach when dealing with small sample sizes, as these authors were, it can also lead to highly inconsistent results and is prone to overfitting [61]. Similarly, 36% of the studied algorithms varied in accuracy by tumor type. On average, these algorithms performed the worst with the classification of ependymoma. This is most attributable to the relative under-representation of ependymoma samples in these unbalanced datasets with 10 studies each featuring fewer than 20 ependymoma samples. While this is not surprising given that ependymomas only represent 8% to 15% of PFTs, the improved representation of rare tumors in these cohorts would improve the overall accuracy of the generated algorithms [62]. Oversampling would provide one potential methodologic solution to address the rare tumor problem. However, algorithm developers must strike a balance to not oversample to an extent that there is overgeneralization of the minority class [63].

Potentially the most actionable limitations relate to data use and method reporting. Most of the included studies (76%) were retrospective, which limits generalizability. Authors frequently used incomplete radiographic or molecular data as inputs. While maintaining a low bar for data inclusion increases sample sizes and generalizability, accuracy would be improved if only complete cases were included. Nine papers additionally lacked sufficient detail in their methods to determine the training or validation set size, number of clinical sites involved, or method of feature extraction [22,23,26,27,32,35,38,39,40]. Machine learning approaches are commonly critiqued as being “black boxes” to their users [64]. The ambiguous definition of the methods and inconsistent reporting of performance metrics serves to further reinforce this criticism and will continue to impede progress if changes are not made.

### 4.5. Posterior Fossa Algorithm Recommendations of Best Practice

We make the following suggestions of best practices for the development of PFT classification algorithms based on our analysis of the algorithm performance and limitations. From a procedural standpoint, most algorithms followed the commonly accepted framework of image acquisition, normalization, feature extraction, dimensionality reduction through feature selection, and classification [65]. Preprocessing and filtering prior to extraction increase the resolution of the extracted imaging features and the subsequent dimensionality reduction removes noise and random error, increasing accuracy [34,65,66,67]. The majority of the classification algorithms identified in this review applied such techniques, which partially explains the high accuracies reported across many algorithms. This process should continue to be employed. While radiomics-based MLAs have classically applied a single classifier on one set of inputs, Zhang et al. [33] highlighted the value of ensemble classifiers that can identify the combination of models with the highest efficacy. As an individual algorithm’s efficacy varied by tumor type, it is necessary to trial multiple combinations of classifiers to identify the ideal system for the specific problem [17,19,20,21,22,23,26,27,28,29,30,35,37,68].

Additionally, algorithms should be trialed prospectively in the clinic with large training and validation sets that equally represent all included tumor types. This recommendation holds especially true for ependymoma, which, while rare, was consistently under-represented in the PFT cohorts being analyzed [16,17,18,21,27,30]. While no one-size-fits-all cohort size can be recommended, a minimum sample size should be chosen to ensure that results are adequately powered. In situations where available data are limited, such as with ependymoma, other machine learning methods can be employed, including model pretraining, semi-supervised learning, or self-supervised learning [69,70]. Standardized results reporting is also necessary to facilitate algorithm comparison and assessment. Each study should report, at minimum, AUC, accuracy, sensitivity, and specificity on both an aggregated as well as a per-tumor basis. One approach to ensure standardized performance analysis involves the curation of a benchmark dataset on which different models can be compared fairly and reproducibly, as has already been implemented with other radiographic data [71,72]. Such a dataset should be derived from multiple centers and contain representative and balanced data, with clear training, validation, and testing subsets. Finally, to address the concern for poor transparency in algorithm development and function the following steps can be taken: (1) local features can be aggregated to give a sense of the overall model, (2) methods such as the “predictive, descriptive, relevant” framework described by Murdoch et al. [73] or the NTRPRT guideline developed by Chen et al. [74] can be utilized to ensure that algorithms are maximally interpretable, and (3) uncertainty measures can be included in model predictions to flag when the model is prone to misclassifications and highlight when human intervention may be required [75,76].

Algorithms developed from MR imaging, microscopy slides, and molecular data were all similarly efficacious [17,21,38,39,40]. While algorithms that improve the cost or speed of tissue diagnosis still have clinical value, algorithms developed from imaging data should be prioritized as computed tomography (CT) and MR are significantly less invasive than tissue collection.

### 4.6. Limitations & Future Directions

This systematic review has some limitations. Papers were only sampled through 31 July 2022, so any additional algorithms classifying PFTs published since have not been included. While potentially clinically useful, this analysis excluded algorithms classifying PFTs by molecular subtypes or prognosis to facilitate the easy comparison of identified algorithms. Algorithm critiques were based solely on the published description of the algorithm at the time this paper was written. Additional data or documentation covering algorithm operation or performance published elsewhere may not be included in this analysis.

The algorithms reported in this paper offer many different approaches to the classification and diagnosis of PFTs based on imaging or molecular features. While some of these methods are compared to a clinical standard, such as a neuroradiologist, many are not. Additional work is needed to make these comparisons to the standard of care and, more importantly, to study the efficacy of these algorithms in the clinical environment. It is postulated that the true clinical integration of machine learning will manifest as a symbiosis between the physician and the developed algorithms instead of the algorithm replacing the physician [77]. Thus, further work is also needed to investigate how physicians interact with these algorithms and how neuroradiologists or neuropathologists can apply these methods to further improve diagnostic accuracy. As previously discussed, a significant barrier to the clinical implementation of machine learning classification algorithms is the methodologic limitations in algorithm design and testing. While proposed solutions are resource-intensive, they seek to make this complex technology more digestible to the typical physician, who is not well-versed in machine learning methods. Multi-institutional collaborations in the field could allow for resource pooling, access to larger sample sizes, and increased exposure of MLAs to industry stakeholders.

## 5. Conclusions

Overall, machine learning has the potential to improve diagnostic speed and accuracy for pediatric PFTs. Developed algorithms focused on the classification of medulloblastoma, pilocytic astrocytoma, and ependymoma with inconsistent results. Individual algorithms reported exceptional performance metrics while others yielded suboptimal outcomes. While a minority of algorithms consistently outperformed the current clinical standard of care, most were nonsuperior or lacked such a comparison. Common limitations include poor methods of reporting, use of small sample sizes, under-representation of certain tumor types such as ependymoma, and methodological limitations inherent to the development of MLAs. The advancement of these algorithms to clinical use will necessitate adherence to consistent data reporting standards, training, and validation in larger sample sizes, prospective trials in real-time clinical workflows, and the study of algorithms as an adjunct to the current standard of care rather than as a replacement.

## Figures and Tables

**Table 1:** Overview of studies applying machine learning to the diagnosis and discrimination of pediatric posterior fossa tumors. Provided is a summary of key study attributes and machine learning methods applied.

Paper	Tumor Type	Imaging/Assay Used	Prospective vs. Retrospective	Study Population	# of Sites	Ground Truth	Training Set	Validation Set	Image Segmentation Method	Normalization Used	Feature Selection Used	Number of Features Extracted	Texture Analysis Employed	Deep Learning Architecture
**Radiographic Algorithms**														
Arle et al., 1997 [16]	AS, EP, PNET	NC-T2MR, MR-spectroscopy	Prospective	10 AS, 7 EP, 16 PNET	1	Histologic diagnosis	150 ^*^	9	Manual	No	No	20	No	NN
Bidiwala et al., 2004 [17]	EP, MB, PA,	CE-T1MR, CE-T2MR	Retrospective	4 EP, 15 MB, 14 PA	1	Histologic diagnosis	32	1 (× 33) ^#^	Manual	Yes	No	36	No	NN
Davies et al., 2022 [18]	EP, MB, PA	NC-T1MR, NC-T2MR, DWI, MR-spectroscopy	Prospective	7 EP, 32 MB, 28 PA	1	Histologic diagnosis	34	33	Manual	Yes	No	19	No	Multivariate classifier w/bootstrap cross-validation
Dong et al., 2021 [19]	EP, MB	CE-T1MR, DWI	Retrospective	24 EP, 27 MB	1	Histologic diagnosis	~46 (90% of cases)	~5 (~10% of cases)	Semi-automatic	Yes	Yes	188	Yes	Adaptive boosting w/3 classifiers: kNN, RF, SVM
Dong et al., 2022 [20]	EP, MB, PA	NC-T1MR, NC-T2MR, CE-T1MR, FLAIR-MR, DWI	Retrospective	32 EP, 67 MB, 37 PA	1	Histologic diagnosis	106	30	Semi-automatic	Yes	Yes	11,958	No	SVM
Fetit et al., 2015 [21]	EP, MB, PA	NC-T1MR, NC-T2MR	Retrospective	7 EP, 21 MB, 20 PA	1	Histologic diagnosis	47	1 (×48) ^#^	Semi-automatic	Yes	Yes	2D—454 3D—566	Yes	6 classifiers: NB, kNN, classification tree, SVM, ANN, LR
Grist et al., 2020 [22]	EP, MB, PA	NC-T1MR, NC-T2MR, CE-T1MR, FLAIR-MR, DWI, DSC-MR	Prospective	10 EP, 17 MB, 22 PA	4	Histologic diagnosis	-	-	Manual	Yes	Yes	Not reported	No	4 classifiers: NN, RF, SVM, kNN
Li et al., 2019 [25]	EP, MB	NC-T1MR, NC-T2MR	Retrospective	58 patients, breakdown unspecified	1	Histologic diagnosis	~41 (70%)	~17 (30%)	Manual	Yes	Yes	300	Yes	Bagging and boosting w/9 classifiers: kNN, SVM, NN, classification and regression trees, RSM, ELM, NB, RF, partial LSR
Li et al., 2020 [24]	EP, PA	NC-T1MR, NC-T2MR	Retrospective	45 patients, breakdown unspecified	1	Histologic diagnosis	~32 (70%)	~13 (30%)	Manual	No	Yes	300	Yes	SVM
Novak et al., 2021 [26]	ATRT, EP, LGT MB, PA	DWI	Retrospective	4 ATRT, 26 EP, 3 LGT 55 MB, 36 PA	5	Histologic diagnosis	-	-	Manual	Yes	Yes	Not reported	No	2 classifiers: NB, RF
Orphanidou-Vlachou et al., 2014 [27]	EP, MB, PA	NC-T1MR, NC-T2MR	Retrospective	5 EP, 21 MB, 14 PA	1	Histologic diagnosis	-	-	Manual	Yes	Yes	279	Yes	2 classifiers: LDA, PNN
Payabvash et al., 2020 [28]	AAS, ATRT, AXA, CPP, EP, GBM, GG, GNT, HB, LGG, lymphoma, MB metastases, PA, SEP	DWI	Retrospective	7 AAS, 6 ATRT, 1 AXA, 4 CPP, 27 EP, 6 GBM, 1 GG, 2 GNT, 44 HB, 10 LGG, 8 lymphoma, 26 MB 65 metastases, 43 PA, 6 SEP	1	Histologic diagnosis	199	49	Manual	Yes	No	24	No	4 classifiers: NB, RF, SVM, NN
Quon et al., 2020 [31]	DMG, EP, MB, PA	NC-T1MR, NC-T2MR, DWI	Retrospective	122 DMG, 88 EP, 272 MB, 135 PA	5	Histologic diagnosis	527 (scans)	212 (scans)	N/A	Yes	No	Not reported	No	Modified ResNet architecture
Rodriguez et al., 2014 [23]	EP, MB, PA	NC-T1MR, NC-T2MR, DWI	Retrospective	7 EP, 17 MB, 16 PA	Multiple	Histologic diagnosis	-	-	Manual	Yes	Yes	183	Yes	SVM
Wang et al., 2022 [29]	EP, MB, PA	NC-T1MR, NC-T2MR, DWI	Retrospective	13 EP, 59 MB, 27 PA	1	Histologic diagnosis	70	20	Manual	Yes	Yes	315	Yes	RF
Zarinabad et al., 2017 [32]	EP, MB, PA	NC-T1MR, NC-T2MR, MR-spectroscopy	Retrospective	10 EP, 38 MB, 42 PA	1	Histologic diagnosis	-	-	Automatic w/manual review	No	Yes	17	No	Adaptive boosting w/4 classifiers: NB, SVM, ANN, LDA
Zarinabad et al., 2018 [30]	EP, MB, PA	MR-spectroscopy	Retrospective	4 EP, 17 MB, 20 PA	4	Histologic diagnosis	37	4	Manual	No	Yes	19	No	3 classifiers: LDA, SVM, RF
Zhang et al., 2021 [34]	ATRT, MB	CE-T1MR, NC-T2MR	Retrospective	48 ATRT, 96 MB	7	Histologic diagnosis	108	36	Manual	No	Yes	1800	Yes	Extreme gradient boosting w/5 classifiers: SVM, LR, kNN, RF, NN
Zhang et al., 2021 [35]	EP, MB, PA	CE-T1MR, CE-T2MR	Retrospective	97 EP, 274 MB, 156 PA	Multiple	Histologic diagnosis	395	132	Manual	No	Yes	1800	No	Extreme gradient boosting w/5 classifiers: SVM, LR, kNN, RF, NN
Zhang et al., 2022 [33]	EP, HGG, SET	CE-T1MR, NC-T2MR	Retrospective	54 EP, 127 HGG, 50 SET	7	Histologic diagnosis	173	58	Manual	Yes	Yes	1800	Yes	Extreme gradient boosting w/binary and single-stage multiclass classifier: SVM, LR, kNN, RF, NN
Zhao et al., 2022 [36]	EP, MB, PA	CE-T1MR, NC-T2MR, DWI, MR-spectroscopy	Prospective	17 EP, 48 MB, 60 PA	4	Histologic diagnosis	-	116	Manual	Yes	Yes	15	No	5 classifiers: NB, LDA, SVM, kNN, multinomial log-linear model fitting via NN
Zhou et al., 2020 [37]	EP, MB, PA	CE-T1MR, NC-T2MR, DWI	Retrospective	70 EP, 111 MB, 107 PA	4	Histologic diagnosis	202	86	Manual	Yes	Yes	3087	Yes	Used tree-based pipeline optimization tool to find optimal architecture using 8 classifiers w/bagging and boosting: NN, decision tree, NB, RF, SVM, LDA, kNN, generalized linear models
**Molecular Algorithms**														
Danielsson et al., 2015 [38]	EP, ETMR, DIPG, GBM, MB, PA	Illumina 450K methylation array data	Retrospective	48 EP, 10 ETMR, 28 DIPG, 178 GBM, 238 MB, 58 PA	Multiple	Histologic diagnosis	472	18, 28 separately	N/A	No	Yes	900	No	3 classifiers: RF, LDA, stochastic generalized boosted models
Hollon et al., 2018 [39]	AS, chordoma, CPP, DMG, EP, ET, germinoma, GG, HB, MB, PA	Microscope slides	Prospective	33 patients, breakdown unspecified	1	Histologic diagnosis	25	-	N/A	Yes	No	13	No	RF
Leslie et al., 2012 [40]	AS, EP, GG, MB, ODG, other glioma	Microscope slides	Prospective	23 patients, breakdown unspecified	1	Histologic diagnosis	-	-	N/A	Yes	Yes	Variable by tumor type	No	SVM

AAS, anaplastic astrocytoma; ANN, artificial neural network; ATRT, atypical teratoid rhabdoid tumor; AXA, anaplastic xanthoastrocytoma; CPP choroid plexus papilloma; DMG, diffuse midline glioma; ELM, extreme learning machine; EP, ependymoma; ET, embryonal tumor; ETMR, embryonal tumors with multilayered rosettes; GBM, glioblastoma multiforme; GG, ganglioglioma; GNT, glioneural tumor; HB, hemangioblastoma; HGG, high-grade glioma; kNN, k-nearest neighbor; LDA, linear discriminant analysis; LGG, low-grade glioma; LR, logistic regression; LSR, least square regression; MB, medulloblastoma; NB, naïve Bayesian; NN, neural network; ODG, oligodendroglioma; PA, pilocytic astrocytoma; PNN, probabilistic neural network; RF, random forest; RSM, random subspace method; SEP, subependymoma; SET, supratentorial embryonal tumor; SVM, support vector machine. * Samples created from original data. ^#^ One case was withheld from the training set and used for validation.

**Table 2:** Summary of common machine learning classifiers used in the classification of posterior fossa tumors.

Classifier Algorithm	Description
K-nearest neighbor	Determines the probability a datapoint will fall into a group based on its distance from the group’s members
Support vector machine	Assigns datapoints to one of two or more categories based on their locations on a space where the distance between the categories is maximized
Neural network	Infers the category of input data through layers of weighted non-linear or linear operations
Extreme learning machine	A feedforward neural network method with faster convergence
Classification tree	Divides datapoints into categories based on the homogeneity of independent variables
Regression tree	Divides data by iteratively partitioning independent variables to minimize mean square error
Random forest	An ensemble method that aggregates outputs of regression trees or classification trees
Naïve Bayes	Applies Bayes’ theorem to classify datapoints by independently considering the value of each independent variable
Partial least square regression	Identifies a subset of independent variables as significant predictors and then runs a regression with these predictors
Linear discriminant analysis	Identifies a linear combination of independent variables that divides datapoints into categories

**Table 3:** Summary of general performance metrics for algorithms developed to discriminate between common pediatric posterior fossa tumors.

Study	AUC	Accuracy	Sensitivity	Specificity
**Discrimination of EP vs. MB**				
Dong et al., 2021 [19]	0.75–0.91	68.6–86.3	-	-
Li et al., 2019 [25]	-	74.6–85.4	-	-
Zhang et al., 2021 [35]	0.92	87.2	91.9	70.0
**Discrimination of EP vs. PA**				
Li et al., 2020 [24]	0.87–0.88	87.0–88.0	90.0–93.0	80.0–83.0
**Discrimination of EP vs. MB vs. PA**				
Bidiwala et al., 2004 [17]	-	-	72.7–85.7	86.4–92.9
Dong et al., 2022 [20]	0.94–0.98	80.0–84.9	80.0–84.9	-
Fetit et al., 2015 [21]	0.81–0.99	71.0–92.0	-	-
Grist et al., 2020 [22]	-	50.0–85.0	-	-
Novak et al., 2021 [26]	-	84.6–86.3	-	-
Orphanidou-Vlachou et al., 2014 [27]	-	37.5–93.8	-	-
Rodriguez et al., 2014 [23]	-	75.2–91.4	-	-
Wang et al., 2022 [29]	-	93.8	-	-
Zarinabad et al., 2018 [30]	-	81.0–86.0	-	-
Zarinabad et al., 2017 [32]	-	80.0–93.0	-	-
Zhang et al., 2021 [35]	0.90	82.6–94.5	73.9–91.8	86.9–95.9
Zhao et al., 2022 [36]	-	84.0–88.0	-	-
Zhou et al., 2020 [37]	0.91–0.92	74.0–83.0	-	-

AUC, area under the curve; EP, ependymoma; MB, medulloblastoma; PA, pilocytic astrocytoma.

**Table 4:** Summary of the reported diagnostic accuracies of commonly employed machine learning algorithms for posterior fossa tumors.

Algorithm	Accuracy (Mean +/− SD)			
	Overall	EP	MB	PA
PNN	89.7 +/− 3.8	-	-	-
Naïve Bayes	85.7 +/− 2.5	87.4 +/− 6.3	88.9 +/− 4.3	90.7 +/− 3.5
LR	82.5 +/− 7.5	85.4 +/− 11.2	85.5 +/− 9.5	88.6 +/− 8.4
ANN	82.5 +/− 13.4	91.5 +/− 4.9	88.5 +/− 10.6	86.5 +/− 13.4
Classification tree	79.0 +/− 5.7	90.0 +/− 7.1	87.5 +/− 3.5	82.0 +/− 4.2
SVM	78.2 +/− 10.7	84.3 +/− 7.1	88.7 +/− 5.9	90.5 +/− 7.0
RF	77.7 +/− 12.3	81.6 +/− 12.0	93.6 +/− 1.3	95.8 +/− 5.8
kNN	69.4 +/− 13.1	86.2 +/− 6.2	87.5 +/− 7.3	85.5 +/− 6.4
LDA	60.5 +/− 21.4	-	-	-

Diagnostic accuracies are reported as the mean +/− the standard deviation (SD) of all reported accuracies for each machine learning classifier. Both global and tumor-specific accuracies are reported. A ‘-’ indicates that no data were available on the diagnostic accuracy of the specified algorithm for the specified tumor type. ANN, artificial neural network; EP, ependymoma; *k*NN, k-nearest neighbor; LDA, linear discriminant analysis; LR, logistic regression; MB, medulloblastoma; PA, pilocytic astrocytoma; PNN, probabilistic neural network; RF, random forest; SVM, support vector machine.

**Table 5:** Common limitations of machine learning algorithms for the classification of posterior fossa tumors.

Limitation	N (%)
Retrospective data collection	19 (76%)
Small training or validation sets	18 (72%)
Unequal distribution of tumor types in training cohorts	17 (68%)
Methods lacking sufficient detail	9 (36%)
Performance varies significantly by tumor type	9 (36%)
Institutional differences in imaging/molecular acquisition	8 (32%)
No inclusion of relevant clinical variables	6 (24%)
Training and validation completed on the same dataset	4 (16%)

## Supplementary Materials

The following supporting information can be downloaded at: https://www.mdpi.com/article/10.3390/cancers14225608/s1, Table S1: Summary of performance metrics for machine learning algorithms that discriminate between both common and rare posterior fossa tumors.
