# Supplementary material for: Machine Learning in the Classification of Pediatric Posterior Fossa Tumors: A Systematic Review
Source: Cancers (Basel). 2022 Nov 15;14(22):5608. doi: 10.3390/cancers14225608 (PMC9688156; doi:10.3390/cancers14225608)
Supplement: Supplementary file 1 [file cancers-14-05608-s001.zip › cancers-1971433-supplementary.pdf]

Supplementary Materials：

Study	AUC	Accuracy	Sensitivity	Specificity
**Discrimination of AS vs. EP vs. PNET**				
Arle et al. 1997[16]	-	58.5-94.6	-	-
**Discrimination of AS vs. chordoma vs. CPP vs. DMG vs. EP vs. ET vs. germinoma vs. GG vs. HB vs. MB vs. PA**				
Hollon et al. 2018[39]	0.96-0.97	89.4-100.0	86.5-90.3	92.3-95.7
**Discrimination of AS vs. EP vs. GG vs. MB vs. ODG vs. other glioma**				
Leslie et al. 2012[40]	-	95.9	-	-
**Discrimination of EP vs. DMG vs. MB vs. PA**				
Quon et al. 2020[31]	-	92.0	-	-
**Discrimination of ATRT vs. MB**				
Zhang et al. 2021[34]	0.73-0.86	81.0-83.0	80.0	82.0-91.0
**Discrimination of DIPG vs. EP vs. ETMR vs. GBM vs. MB vs. PA**				
Danielsson et al. 2015[38]	-	98.3	-	-

Supplementary Table S1: Summary of performance metrics for machine learning algorithms that discriminate between both common and rare posterior fossa tumors.

AS, astrocytoma; ATRT, atypical teratoid rhabdoid tumor; AUC, area under the curve; CPP choroid plexus papilloma; DMG, diffuse midline glioma; EP, ependymoma; ET, embryonal tumor; ETMR, embryonal tumors with multilayered rosettes; GBM, glioblastoma multiforme; GG, ganglioglioma; HB, hemangioblastoma; MB, medulloblastoma; ODG, oligodendroglioma; PA, pilocytic astrocytoma.

Searches

Pubmed [7/31/2022, 106 results]

(((machine learning) OR (artificial intelligence)) AND ((glioma) OR (hemangioblastoma) OR (dermoid) OR (gangliocytoma) OR (rhabdoid) OR (teratoid) OR (posterior fossa) OR (ependymoma) OR (medulloblastoma) OR (pilocytic astrocytoma)) AND (pediatric))

Web of Science [7/31/2022, 44 results]

(((machine learning) OR (artificial intelligence)) AND ((glioma) OR (hemangioblastoma) OR (dermoid) OR (gangliocytomas) OR (rhabdoid) OR (teratoid) OR (posterior fossa) OR (ependymoma) OR (medulloblastoma) OR (pilocytic astrocytoma)) AND (pediatric))

EMBASE [7/31/2022, 282 results]

('machine learning'/exp OR 'machine learning' OR (('machine'/exp OR machine) AND ('learning'/exp OR learning)) OR 'artificial intelligence'/exp OR 'artificial intelligence' OR (artificial AND ('intelligence'/exp OR intelligence))) AND ('glioma'/exp OR glioma OR 'hemangioblastoma'/exp OR hemangioblastoma OR

'dermoid'/exp OR dermoid OR 'gangliocytoma'/exp OR gangliocytoma OR rhabdoid OR teratoid OR 'posterior fossa'/exp OR 'posterior fossa' OR (posterior AND fossa) OR 'ependymoma'/exp OR ependymoma OR 'medulloblastoma'/exp OR medulloblastoma OR 'pilocytic astrocytoma'/exp OR 'pilocytic astrocytoma' OR (pilocytic AND ('astrocytoma'/exp OR astrocytoma))) AND ('pediatric'/exp OR pediatric)
